# Early prediction of clinical scores for left ventricular reverse remodeling using extreme gradient random forest, boosting, and logistic regression algorithm representations

**DOI:** 10.3389/fcvm.2022.864312

**Published:** 2022-08-17

**Authors:** Lu Liu, Cen Qiao, Jun-Ren Zha, Huan Qin, Xiao-Rui Wang, Xin-Yu Zhang, Yi-Ou Wang, Xiu-Mei Yang, Shu-Long Zhang, Jing Qin

**Affiliations:** ^1^Heart Centre, Affiliated Zhongshan Hospital of Dalian University, Dalian, China; ^2^School of Software Engineering, Dalian University, Dalian, China; ^3^Medical College, Dalian University, Dalian, China

**Keywords:** left ventricular reverse remodeling, heart failure with reduced ejection fraction, prediction model, machine learning, heart failure

## Abstract

**Objective:**

At present, there is no early prediction model of left ventricular reverse remodeling (LVRR) for people who are in cardiac arrest with an ejection fraction (EF) of ≤35% at first diagnosis; thus, the purpose of this article is to provide a supplement to existing research.

**Materials and methods:**

A total of 109 patients suffering from heart attack with an EF of ≤35% at first diagnosis were involved in this single-center research study. LVRR was defined as an absolute increase in left ventricular ejection fraction (LVEF) from ≥10% to a final value of >35%, with analysis features including demographic characteristics, diseases, biochemical data, echocardiography, and drug therapy. Extreme gradient boosting (XGBoost), random forest, and logistic regression algorithm models were used to distinguish between LVRR and non-LVRR cases and to obtain the most important features.

**Results:**

There were 47 cases (42%) of LVRR in patients suffering from heart failure with an EF of ≤35% at first diagnosis after optimal drug therapy. General statistical analysis and machine learning methods were combined to exclude a number of significant feature groups. The median duration of disease in the LVRR group was significantly lower than that in the non-LVRR group (7 vs. 48 months); the mean values of creatine kinase (CK) and MB isoenzyme of creatine kinase (CK-MB) in the LVRR group were lower than those in the non-LVRR group (80.11 vs. 94.23 U/L; 2.61 vs. 2.99 ng/ml; 27.19 vs. 28.54 mm). Moreover, AUC values for our feature combinations ranged from 97 to 94% and to 87% when using the XGBoost, random forest, and logistic regression techniques, respectively. The ablation test revealed that beats per minute (BPM) and disease duration had a greater impact on the model’s ability to accurately forecast outcomes.

**Conclusion:**

Shorter disease duration, slightly lower CK and CK-MB levels, slightly smaller right and left ventricular and left atrial dimensions, and lower mean heart rates were found to be most strongly predictive of LVRR development (BPM).

## Introduction

Angiotensin-converting enzyme inhibitors (ACEI), angiotensin II receptor blockers (ARB), β-blockers, and mineralocorticoid receptor antagonists (MRA) are considered the cornerstone for the treatment of patients suffering from heart failure with reduced ejection fraction (HFrEF), as these approaches can facilitate left ventricular reverse remodeling (LVRR), minimize the hospital admission rate of patients with heart failure (HF), and decrease the overall rates of mortality and cardiovascular mortality, including those related to sudden cardiac death (SCD) (1–5). In patients with HFrEF, a guideline-directed drug therapy has the potential to improve the left ventricle function and counteract the frequently observed adverse cardiac remodeling. Therefore, predicting LVRR is essential for the development of long-term treatment strategies, which include the use of an implantable cardioverter defibrillator (ICD), the use of a left ventricular assist device (LVAD), cardiac resynchronization therapy (CRT), and heart transplantation (HTx). While evaluation of these predictive factors is straightforward, using only individual predictive factors is inadequate and can lead to inaccuracy. Limited information is available on factors predicting the improvement in left ventricular ejection fraction (LVEF) by implementing drug therapy in patients with HFrEF (6, 7), and LVRR usually occurs within 1–2 years in patients who experience heart attacks (8). ICD implantation is recommended as the primary form of prevention in clinical guidelines only when the LVEF is re-evaluated to be 35% again in patients after at least 3–6 months of receiving optimal drug therapy (9). Therefore, if we can identify LVRR in combination with several common clinical diagnosis methods in patients with an EF of ≤35% (HFrEF) at first diagnosis, this will aid in determining whether ICD implantation should be performed to prevent sudden cardiac death. Compared with traditional statistical methods, machine learning methods are more objective and effective in feature selection and the processing of possible non-linear variables. The representative algorithms include extreme gradient boosting (XGBoost) and random forest. XGBoost, a machine learning algorithm first proposed by Professor Tianqi Chen, improves on the gradient-boosted decision tree (GDBT) algorithm (10) and has been widely used in other machine learning competitions, such as Kaggle (11). This method has also been successfully applied in disease diagnosis (12, 13) and health risk prediction (14–17). Therefore, the tree-based integrated learning method—also known as the extreme gradient boosting method—was used to investigate and analyze medical data, establish a prediction model, and validate the effectiveness of the algorithm.

## Materials and methods

### Study population

This study was a one-center retrospective research study. Clinical data were collected from 265 consecutive inpatients who were first diagnosed with an EF of ≤35% (HFrEF) in the Affiliated Zhongshan Hospital of Dalian University from March 2018 to March 2020, with multiple follow-up records collected for each patient. Patients suffering from cardiomyopathy with possible spontaneous LVRR, such as patients suffering from tachycardia-induced cardiomyopathy, perinatal cardiomyopathy, myocarditis, and alcoholic dilated cardiomyopathy, were excluded (5). Patients suffering from atrial fibrillation (AF) with a resting heart rate > 100 BPM were excluded because they may have developed tachycardia-induced cardiomyopathy (18). Patients with ventricular remodeling that was improved or worsened by medical or surgical interventions (apart from optimal drug therapy for HF) were also excluded; these patients had undergone coronary revascularization or CRT within the prior 2 months. Moreover, patients were excluded if they were found to have an ICD or severe organic heart valvular disorder, were currently on a targeted dose or a maximum tolerated dose of drug therapy for HF, or had experienced a myocardial infarction within the prior 2 months. The research was officiated by the institutional evaluation board of the Affiliated Zhongshan Hospital of Dalian University and carried out according to the ethical standards set out in the Declaration of Helsinki (1964) and its subsequent protocol alterations. Well-versed consent was not required because participation in our research was anonymous. All patients were treated with standard drugs outlined in the current guidelines.

### Data collection

Patient baseline assessment included blood samples for laboratory examination, ECG, physical examination, and detailed clinical history. The period of heart attack was defined as the time in months from the signs of heart failure onset (or left ventricular systolic dysfunction, and in the absence of symptoms) to enrollment in the study, while the onset of symptoms of heart failure was defined as the change of NYHA classification in patients’ daily lives. The final echocardiogram taken prior to study commencement was considered the patient’s basic echocardiography.

The drug therapy for HF was optimized in the ward based on clinical guidelines following baseline assessment (2). The treatment protocol typically consisted of drugs beneficial to left ventricular remodeling and disease-modifying agents (β-blockers, ACEI/ARBs, MRAs) that were administered until the target dose or maximum tolerated dose could be achieved. The follow-up assessment, including clinical history, physical examination, ECG, and echocardiography, was performed 12 months later.

### Echocardiography

A technician attached to the cardiovascular ultrasound department performed all echocardiography according to international guidelines. The left ventricular end-diastolic width was fitted in the para long-axis M-mode guided by the 2D echo. In the sinus rhythm, the left ventricular volume was measured at apex four and two ventricles using the Simpson method. In the non-sinus rhythm, the left ventricular mass was measured in five cycles and then averaged. The left ventricular end-diastolic diameter and the capacity were indexed by body surface area. LVEF was obtained with reference to the changes in the left ventricular volume.

### Return visit

Follow-up appointments for patients were made as required. The visits ended in February 2021, upon heart transplantation, or on the date of death. Transthoracic echocardiography was performed during all visits. LVRR was defined as an absolute raise in LVEF from ≥10% to a final value of >35%, lasting up to the final visit. Non-LVRR was well-defined as a total upsurge in the left ventricular ejection portion of <10% or with a final value of <35%, or a reduction in the left ventricular end-diastolic diameter of <10% for all visits except for those less than 1 year. Patients who failed to meet the criteria of LVRR and had the final visit within 1 year were excluded.

### Statistical study

The data were presented as the mean normal deviation for generally distributed variable quantities and median SD for those with a non-normal distribution (numerical range). Heterogeneity in the variance was discovered by means of a left rotation test, which had an alpha value of <0.05. Different groups were classified using the Mann–Whitney *U* test and the chi-square test, which are autonomous *t*-tests.

### Data input

A total of 53 samples were collected for examination; details are presented in Table 1. In addition, there were seven features with missing data, representing a missing data rate lower than 10%. The missing variables were filled by median data, and all data were subject to min-max normalization according to the following formula (where x stands for the current variable, x_*max*_ for the extreme value of a variable, and x_*min*_ for the smallest value of a variable):

**TABLE 1:** Characteristics of patients grouped by left ventricular reverse remodeling.

Characteristics	LVRR (*n* = 47)	Non-LVRR (*n* = 62)	*P*-value
Duration of disease (months), M (min-max)	7 (0.1–60)	48 (1–168)	<0.001
Female, n (%)	17 (36.17)	23 (37.10)	0.844
Age (years), M (min-max)	65 (29–91)	72 (25–86)	0.013
Smoke, n (%)	16 (34.04)	13 (20.97)	0.004
Alcoholism, n (%)	13 (27.66)	15 (24.20)	0.422
PCI, n (%)	12 (25.53)	15 (24.20)	0.752
Coronary heart disease, n (%)	25 (53.19)	26 (41.93)	0.379
Myocardial infarction, n (%)	5 (10.64) (Missing 1)	6 (9.68)	0.689
Mitral regurgitation, n (%)	11 (23.40)	24 (38.71)	0.002
Hypertension, n (%)	36 (76.60)	38 (61.29)	0.001
Systolic pressure (mmHg), M ± SD	131.3 ± 25.91 (Missing 3)	128.52 ± 26.19	0.986
Diastolic pressure (mmHg), M ± SD	80.07 ± 14.21 (Missing 3)	79.05 ± 14.78	0.911
NYHA, M (min-max)	3 (1–4)	4 (3–4) (Missing 1)	0.003
ACEI/ARB/ARNI, n (%)	34 (72.34) (Missing 1)	43 (69.35) (Missing 7)	<0.001
β-blocker, n (%)	39 (82.98)	36 (58.10)	<0.001
Diuretic, n (%)	37 (78.72)	42 (67.74)	0.100
NT-proBNP (pg/ml), M (min-max)	2464 (31.5–22702) (Missing 2)	5840 (420–35000)	0.000
cTnI (ng/ml), M (min-max)	0.054 (0.002–0.8) (Missing 1)	0.043 (0.012–3.63)	0.013
CK (U/L), M ± SD	80.11 ± 59.08	94.23 ± 56.56	0.865
CK-MB (ng/ml), M ± SD	3.61 ± 5.17	2.99 ± 4.49	0.124
D-Dimer (ug/ml), M ± SD	1.02 ± 1.32	1.27 ± 1.77	0.194
PT%, M ± SD	84.23 ± 18.88	66.58 ± 25.08	0.151
PT (s), M ± SD	14.29 ± 7.13	19.99 ± 15.43	0.045
APTT (s), M ± SD	30.06 ± 7.34	32.36 ± 7.66	0.846
Creatinine (umol/L), M ± SD	102.20 ± 49.10	111.80 ± 79.45	0.369
Urea (mmol/L), M ± SD	12.17 ± 13.41	11.52 ± 7.63	0.322
ALT (IU/L), M ± SD	95.90 ± 510.19	70.71 ± 252.47	0.367
AST (IU/L), M ± SD	118.45 ± 618.67	79.89 ± 256.99	0.304
Triglyceride (mmol/L), M ± SD	1.88 ± 1.64	4.56 ± 25.76	0.149
Total cholesterol (mmol/L), M (min-max)	3.9 (1.21–7.3)	4 (0.33–5.42)	0.001
High density lipoprotein (mmol/L), M ± SD	1.03 ± 0.41	1.00 ± 0.31	0.092
Low density lipoprotein (mmol/L), M (min-max)	2.1 (0.89–5.03)	2.18 (0.81–3.5)	0.003
White blood cell (*10^9^/L), M ± SD	8.46 ± 5.93	6.82 ± 3.10	0.356
Hemoglobin (g/L), M ± SD	132.45 ± 26.58	131.45 ± 24.02	0.444
Platelet (*10^9^/L), M ± SD	195.85 ± 71.87	171.03 ± 68.62	0.660
T_3_ (pmol/L), M ± SD	3.86 ± 0.88	3.59 ± 0.76	0.255
T_4_ (pmol/L), M ± SD	15.30 ± 2.94	15.48 ± 3.60	0.200
Thyroid stimulating hormone (uIU/ml), M ± SD	3.00 ± 3.22	2.11 ± 1.64	0.044
Right ventricular diameter (mm), M ± SD	27.19 ± 5.70	28.54 ± 8.00 (Missing 1)	0.191
LvEd (mm), M ± SD	58.28 ± 8.97	65.03 ± 7.77	0.305
LvSd (mm), M (min-max)	10 (7–53)	10 (8–16)	0.008
Lvpwd (mm), M ± SD	10.49 ± 1.80	9.11 ± 1.44	0.291
La diameter (mm), M (min-max)	45 (10–60)	51 (32–70)	<0.001
bpm, M ± SD	69.94 ± 22.42	88.19 ± 20.32	0.576
PR (ms), M (min-max)	130 (100–256)	160 (140–240)	0.004
QRS (ms), M (min-max)	120 (76–200)	110 (90–194)	0.039
QTc (ms), M ± SD	447.81 ± 45.22	479.31 ± 76.62	0.495
End left, n (%)	1 (2.13)	7 (11.29)	0.000
End right, n (%)	2 (4.26)	3 (4.84)	0.775
Ventricular tachycardia, n (%)	8 (17.02)	7 (11.29)	0.090
Atrial fibrillation, n (%)	12 (25.53)	24 (38.71)	0.004
Atrial flutter, n (%)	2 (4.26)	0 (0.00)	0.001
Dilated cardiomyopathy, n (%)	7 (14.89)	11 (17.74)	0.430

M ± SD, mean ± standard deviation; M (min-max), median (numerical range); PCI, Percutaneous Coronary Intervention; NYHA, New York Heart Association; T_3_, Triiodothyronine; T_4_, Thyroxine; ACEI/ARB/ARNI, Angiotensin converting enzyme inhibitors/angiotensin II receptor blockers/Angiotensin Receptor Neprilysin Inhibitor. *Means multiply (×).


$$x^{*}=\frac{x-x_{\max}}{x_{\max}-x_{\min}}$$

### Model research

**FIGURE 1:**
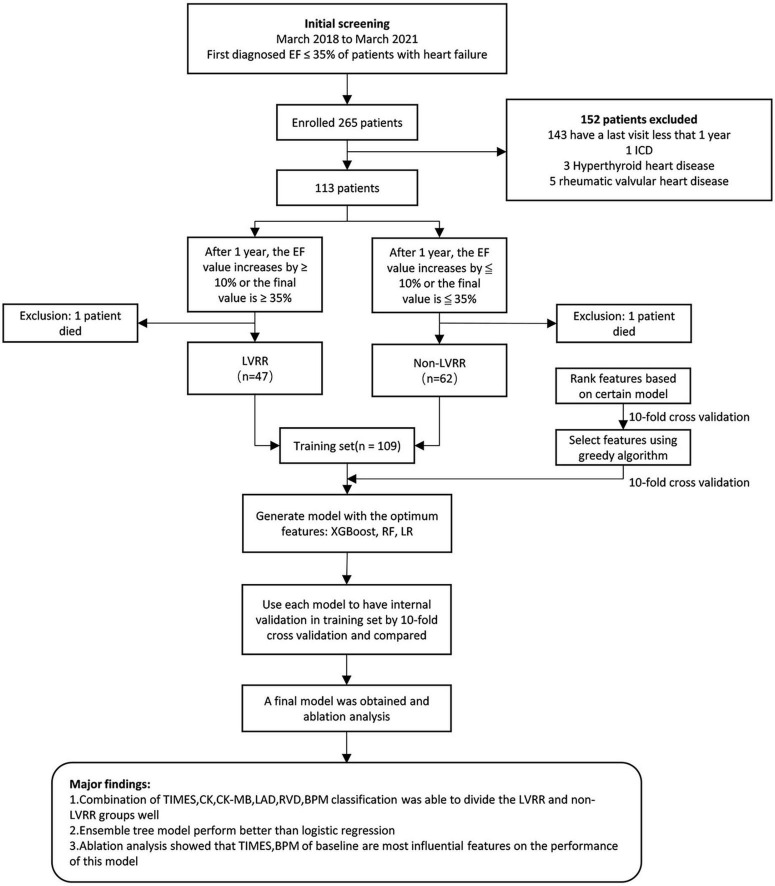
Major results of the research and overall flowchart. EF, left ventricular ejection fraction; LVRR, left ventricular reverse remodeling; XGBOOST, extreme gradient boosting; LR, logistic regression; RF, random forest.

In order to conduct a model research, we used XGBoost, random forest, and logistic regression (19). XGBoost and random forest are both integrated algorithms that can achieve better learning performance than single-learner algorithms. The data were arbitrarily separated into 10 subsets *via* 10-fold cross-authentication. Nine of these subsets were used for training, while one was used for testing; this process was carried out a total of 10 times to guarantee that each subset was tested. Finally, all results were averaged to measure the model performance. The specific experimental process is illustrated in Figure 1. The feature combinations that could reach the highest area under the curve (AUC) value were selected, and the model training was conducted on this basis; this was followed by individual tests on each model and the plotting of receiver operating characteristics (ROC) curves. The significance of each individual feature was then measured *via* an ablation analysis, which is the process of setting up a control group by removing a factor and then measuring the importance of that factor based on the subsequent degree of decline in the experimental results.

### Feature selection

Feature assortment was performed to find the optimal feature grouping in the forecast model. The distribution of all 53 features is shown in Table 1. In order to obtain the sets of important characteristics, the tree-based model feature selection method was used in this research, while a greedy algorithm was used for subsequent filtering on the basis of this feature set according to the AUC value (a greedy algorithm is one that always makes the current best choice when solving a problem; i.e., instead of considering the overall optimum, the local optimum solution is always considered).

The selected features were all greater than zero in terms of importance. The greedy search started with an empty set of important features. The selection was performed according to the following iterative process: first, the feature with the highest AUC values to the set of important features was added; then, the next feature that causes the AUC to reach the highest value in the set of the remaining features to the set of important features was found; this process was repeated until there are no remaining features or there is no increase in the AUC value of the set of important features, thus establishing the final prediction model.

### Baseline features

A total of 378 clinical data points were collected from 109 inpatients. The LVRR was identified in 47 (45.2%) of the 109 examined patients (Figure 1). The distribution and characteristics of these patients are presented in Table 1. Patients with LVRR were prone to elevated systolic pressure, elevated platelet count, decreased serum D-dimer levels, elevated high-density lipoprotein cholesterol (HDL-C), decreased left atrial diameter, decreased right ventricular end-diastolic diameter, and a lower possibility of severe mitral regurgitation (MR). Blockers and ACEIs/ARBs/ARNIs did not differ significantly between the two clusters in terms of their use or doses.

### Statistical tools and machine learning

The scikit-learn 0.24.2 software package was used to perform machine learning in the Python 3.9.1 tool environment. Moreover, the SPSS 26.0 software (IBM SPSS Statistics, IBM, Armonk, NY, United States) was used for statistical analysis.

## Results

### Research and validation of the classifier model

**FIGURE 2:**
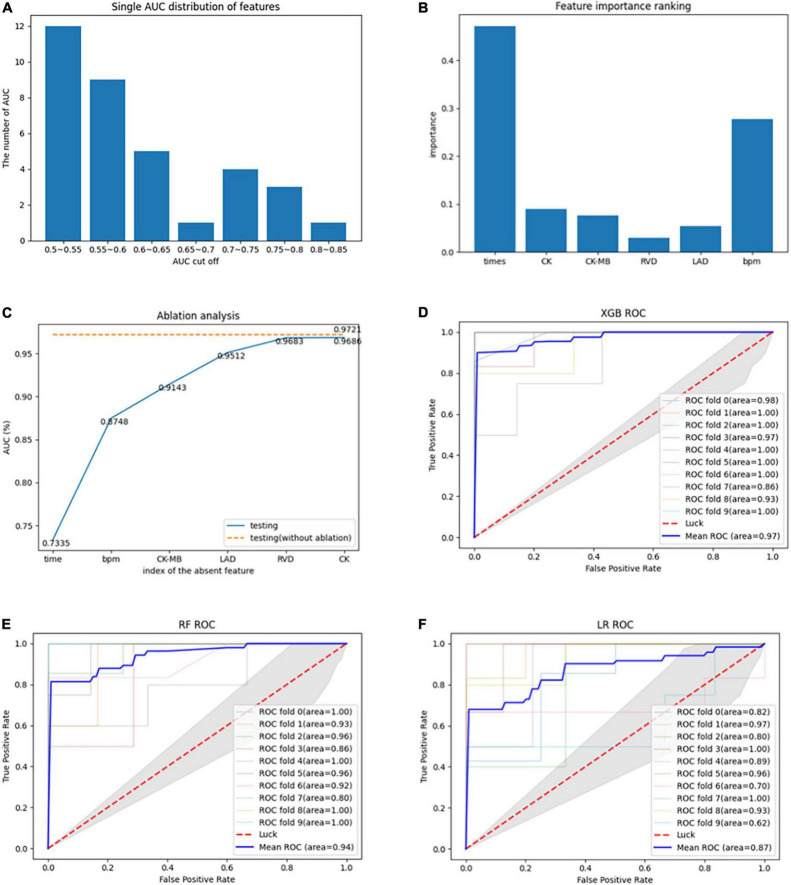
**(A)** AUC values of each feature for classification during the modeling process. **(B)** Importance ranking of the optimal features derived by the greedy algorithm. **(C)** Evaluation of the contribution of each feature to the model through ablation analysis. **(D)** ROC curve of 10-fold cross-validation using extreme gradient boosting algorithm. **(E)** ROC curve of 10-fold cross-validation using random forest algorithm. **(F)** ROC curve of 10-fold cross-validation using logistic regression algorithm.

A total of 35 features were screened out through the tree model. Classification ability tests with or without improvement were performed on each of the 35 features, with the results shown in Figure 2A. Overall, there were 27 (77.14%) features with AUC values less than 0.7 and only eight features with AUC values of 0.7–0.85, with the highest AUC value of 0.82 at the time of disease. Due to the small amount of experimental data available, it was necessary to consider the sensitivity of the current experimental data to particular features; therefore, it was also necessary to validate whether a mixture of multiple features could improve the model performance. The details of the feature selection process are provided in Figure 1. The feature selection was first carried out for the dataset based on the tree model to obtain the important features (only the results of XGBoost are described in this article), resulting in 35 important features being obtained. The greedy algorithm was used to screen the most important feature set from the 35 important features; as a result of this process, six features were identified through screening, with the importance ranking shown in Figure 2B. An average AUC value of 0.97 was finally obtained through the application of these six features to the training XGBoost model under 10-fold cross-validation. This 10-fold cross-validation was also used in the ablation analysis to evaluate the contribution of the six features to model prediction (results are shown in Figure 2C). The absence of any one feature would lead to a decline in the AUC value; here, the observed duration of disease and BPM was found to be the most important features, and the AUC value decreased to 0.73 when the duration of disease was removed. Moreover, a comparison was also drawn with other machine study methods in aspects of performance of prediction. The ROC curves of biggest gradient boosting, random forest, and logistic reversion are plotted in Figures 2D–F, respectively, while the average AUC values of 0.97, 0.94, and 0.84, respectively, were obtained, indicating that the extreme gradient boosting algorithm produced better results when compared with the logistic regression algorithm. The outcomes of the extreme gradient boosting algorithm were slightly better than those of the random forest algorithm. Meanwhile, the recall rate and sensitivity when distinguishing LVRR from non-LVRR cases were compared for the three classifiers (as shown in Table 2), with the results validating the conclusions above.

**TABLE 2:** Model performance comparison.

Classifier	Classification results	Accuracy	Recall rate	F1 value
XGBoost	LVRR	0.858	0.875	0.856
	Non-LVRR	0.896	0.871	0.875
Random forest	LVRR	0.851	0.861	0.836
	Non-LVRR	0.897	0.896	0.886
Logistic regression	LVRR	0.840	0.657	0.696
	Non-LVRR	0.788	0.899	0.825

Our main findings in this study were as follows (as shown in Table 3): (1) extreme gradient boosting and random forest algorithms, when combined with the collected medical data, were superior to the logistic regression algorithm in terms of predicting the variance between LVRR and non-LVRR cases; (2) the time of disease and the mean heart rate were the most important features; and (3) according to the statistical analysis, when compared with the non-LVRR cluster, the LVRR set had a shorter duration of disease, slightly lower creatine kinase (CK) and MB isoenzyme of creatine kinase (CK-MB) values, slightly lower right ventricular diameters and left atrial diameters, and lower mean heart rates (BPM). According to the ablation analysis results, the duration of disease and BPM made greater contributions to the model prediction.

**TABLE 3:** Comparison of important features obtained by greedy algorithms.

Characteristics	LVRR	Non-LVRR
Duration of disease (months), M[Q]	7 (0.1–60)	48 (1–168)
CK, M ± SD	80.11 ± 59.08	94.23 ± 56.56
CK-MB, M ± SD	2.61 ± 5.16	2.99 ± 4.49
Right ventricular diameter	27.19 ± 5.70	28.54 ± 8.00
Left atrial diameter	45 (10–60)	51 (32–70)
Bpm	69.94 ± 22.42	88.19 ± 20.32

## Discussion

To the best of our knowledge, the present research is the first to use an integrated tree model of machine learning to predict LVRR. To avoid the assumptions that a linear relationship exists between variables and the accuracy of statistical models, these models can be used instead. Classifiers such as the XGBoost and random forest algorithms, both of which were used in our research, predicted LVRR with a comparable degree of accuracy. An improved risk factor management in patients with HFrEF may be made possible through the use of these integrated tree models (12). The most important elements of the model were the duration of the disease, the mean heart rate, the CK, the CK-MB, the right ventricular size, and the left atrial size. Early clinical care and early prevention plan application may be significant for identifying patients who do not respond to drug therapy using these machine classifiers. It is important to highlight that our results differ from those of earlier studies. In one study, it was shown that natriuretic peptides may be useful in predicting LVRR; in another, a large variance in LVRR incidence between men and women was found (20). Natriuretic peptides and sex differences were shown by the findings of our study not to be useful in predicting LVRR. There are several possible explanations for this. For example, NT-proBNP was originally included in the 35 key features assessed by the tree model, based on the clinical dataset employed. The classifier proposed herein sought to find the best combination of features that would maximize AUC. Moreover, because the feature combination containing NT-proBNP scored lower in the greedy algorithm’s secondary screening than any of the other feature combinations, NT-proBNP was eventually dropped from further consideration in this article. In the LVRR group, based on our clinical dataset, there were 17 female patients (36.1%), while 23 female patients (or 37.1%) were found in the non-LVRR group. The machine learning algorithm was able to remove sex differences because the proportions between the two sets of data were so similar. Our study findings revealed that the duration of disease was the most significant contribution to the prediction model. In the early stage of heart failure, the optimization of current drug interventions might slow down the progressive loss of myocardial cells; the continuous disorder of myocardial cell proteome, metabolome, and transcriptome; and the progressive erosion of the natural 3D tissues of the extracellular matrix surrounding the cardiomyocytes; in this way, it can improve the LVEF and stabilize the left ventricular reverse remodeling (21). This view was supported by the observation that most clinical cases of recovered left ventricular function linked to lasting clinical steadiness occurred after transient damage (e.g., energy deficiency or cardiotoxin) rather than permanent or/and long-term injury (e.g., myocardial infarction and genetic abnormalities). Mean heart rate also contributed significantly to the LVRR prediction model, while the incidence rate and mortality rate of cardiovascular disease were associated with an increased mean heart rate (BPM). The association between sustained tachycardia (>100 BPM) and the incidence rate and mortality rate of cardiovascular disease was first demonstrated more than 70 years ago (22). The Framingham study in 1987 showed that an increase in heart rate of 10 BPM was linked with a 14% rise in overall mortality rate in the general populace; in addition, the danger of heart attack was significantly higher in subjects with a baseline heart rate > 80 BPM (23). In clinical practice, reports from large registration agencies showed that more than 50% of patients with heart failure diseases still had a heart rate of >70 BPM following optimal drug therapy, and moreover, that there was a notable increase in both the mortality rate and the number of hospitalizations due to cardiac deterioration if it was beyond the threshold. According to the Italian Network on Heart Failure (IN-HF) Outcome Registry (24), registration information on heart failure in Italy identified 3,755 outpatient chronic heart failure patients who were followed up after 1 year: among these, 53.4% had a heart rate of ≥70 BPM, 29.7% had a rate of ≥75 BPM, and 17.2% had a rate of ≥80 BPM. The use of β-blockers to handle heart failure aimed to reduce the patient heart rate to 70 BPM or lower; while the dose adjustment should have been directed toward achieving this goal, the said goal was unfortunately only achieved in half of the patients.

Two significant clinical parameters of the cardiac structure identified *via* echocardiography were observed in this study. Echocardiography represents the first-line analysis method for people with heart failure. Our findings were the same as those of earlier studies on the prognosis of patients who suffered from heart attack. Heart failure with preserved ejection fraction (HFpEF) is associated with the elevated left atrial pressure and its spread to the pulmonary circulation, while HFrEF may be associated with pre- and post-capillary pulmonary arterial hypertension. Compared with the control group (1.4%/mmHg), patients with HFrEF or HFpEF (0.8–0.9%/mmHg) had a moderately lower vascular elastic dilation coefficient (25); recent research has also revealed that right ventricular dysfunction may be indicative of increased pulmonary arterial pressure (26), which may itself be a progressive phase of ventricular remodeling. Although the right ventricular end-diastolic diameter cannot fully reflect the right ventricular function, it can provide valuable predictive information when combined with extreme remodeling features, such as functional mitral regurgitation and other atrial enlargements. Motoki et al. (27) reported that a severe decline in the right ventricular function was associated with left atrial volume index, left ventricular diastolic dysfunction, decreased left ventricular discharge portion, and standard indices of right ventricular diastolic and systolic dysfunctions (right ventricle S’, E’/A’, right atrial volume index) in 171 patients with HFrEF. The long-term (5-year) adverse events (all-cause mortality, hospitalizations for heart relocation, and heart attack) can be predicted based on right ventricular compliance. In addition, adverse events can also be predicted based on the corrected age, right atrial capacity index, right ventricular end-diastolic volume, and left ventricular ejection fraction. The differences between the LVRR and non-LVRR clusters in terms of right ventricular end-diastolic volume and left atrial end-diastolic volume show that blocking the sympathetic nervous system and the renin–angiotensin–aldosterone system could reverse pulmonary vascular resistance, including pulmonary microvascular remodeling, vasoconstriction, and endothelial dysfunction; thus, reduction in the right ventricular end-diastolic volume and left atrial end-diastolic capacity could increase expulsion fraction, thereby improving the left ventricular ejection function. The left ventricular end-diastolic size was not considered a predictive factor for LVRR because the author considered the duration of disease to be the most important predictive factor for LVRR. Patients with shorter disease duration, for example, had milder left ventricular myocardial fibrosis after early optimization of medication therapy, which neither contributed to left ventricular remodeling nor affected the left ventricular end-diastolic volume. A significant biomarker in diagnosing coronary disorders, including acute myocardial infarction (AMI), is CK-MB. Cardiovascular disease can be diagnosed using CK-MB, along with other biomarkers (such as cardiac troponin), when used in combination. An innovative heart failure risk prediction model based on the multi-index technique and the random forest algorithm (28) was created in China in 2019; this study involved 193 people (80 heart failure victims and 113 gender- and age-matched healthy controls) between June 2017 and December 2017. Cardiac biomarkers and echocardiographic measurements were subjected to a correlation and regression analysis. In individuals with heart failure, the levels of CK-MB, BNP, Gal-3, and sST2 were found to be significantly elevated. For HF, BNP was able to accurately predict the outcome of a test (AUC.956). C-reactive protein, sST2, and gal-3 levels were all found to have moderate diagnostic performance for HF. The average decline in accuracy for BNP was much higher than that for other variables. Despite this, the multi-marker model’s sensitivity and specificity improved, reaching 91.5 and 96.7%, respectively. There was also no significant difference between NT-proBNP and CK and CK-MB diagnosis performance in our investigation; moreover, the prognostic effect of myocardial markers in the LVRR group was rarely reported, according to our findings. In addition, HFrEF is a clinical syndrome that affects multiple organ systems due to a variety of causes. It is important to identify LVRR in a timely fashion so as to achieve accurate management and determine the reasonable timing of ICD implantation. Machine learning applications may represent a powerful solution to this problem. Pérez-Rodon et al. (29) began their study in 2017 by selecting outpatients with LVEF of 35% as observational subjects for a 6-month follow-up study to investigate drug optimization treatment. These authors found that ischemic cardiomyopathy, prolonged duration of heart failure, and larger left ventricular end-diastolic diameter were the three most significant predictors of LVRR loss. Moreover, long-term ischemic cardiomyopathy produces scar tissue, which causes an increase in the width of the left ventricular end-diastolic area that is difficult to treat with medicines. It is suggested that an ICD be implanted as soon as possible in these patients because of their significant risk of sudden death.

## Limitations

There are several limitations of our research. For example, one study found that a scan employing cardiac magnetic resonance (CMR) might better predict the LVRR (30). CMR is indeed considered the gold standard for measuring cardiac ventricles; however, because echocardiography is more readily available in daily clinical practice, we did not include additional information regarding the results of cardiac MRI in this article. The efficacy and sensitivity required to evaluate LVRR have been confirmed in prior research. Second, the definition of LVRR was found to vary substantially throughout the literature (31); as a result, the definition of LVRR provided in this article appears somewhat arbitrary. In addition, we did not conduct sensitivity analyses based on various definitions of LVRR, which may have weakened the findings of this study. Another key issue is that various exclusion criteria were applied in this study in order to ensure better completion of the machine learning and provide a more balanced statistical analysis. As the sample size of the study is currently small, prospective studies with larger samples and external validation will be required in the future to obtain stronger evidence. Furthermore, while this study focuses on factors that may predict an elevated risk of sudden cardiac death (SCD), the nature of retrospective investigations makes it difficult to acquire complete diagnoses for each patient.

## Conclusion

The random forest and XGBoost algorithms were found to perform very well at forecasting LVRR in people with HFrEF. The LVRR of HFrEF could be predicted using a combination of conventional laboratory testing and echocardiographic indices. These machine learning classifiers can aid in achieving precise managing and danger assessment for people with HFrEF.

## Data availability statement

The original contributions presented in this study are included in the article/supplementary material, further inquiries can be directed to the corresponding authors.

## Ethics statement

The studies involving human participants were reviewed and approved by Ethics Committee of Affiliated Zhongshan Hospital of Dalian University. The patients/participants provided their written informed consent to participate in this study.

## Author contributions

LL and CQ: conception and design of the research and writing of the manuscript. HQ, X-RW, X-YZ, Y-OW, and X-MY: acquisition of data. LL, CQ, and J-RZ: analysis and interpretation of the data. J-RZ and JQ: statistical analysis. S-LZ and JQ: critical revision of the manuscript for intellectual content. All authors read and approved the final draft.

## References

[1] Gayat E Arrigo M Littnerova S Sato N Parenica J Ishihara S Heart failure oral therapies at discharge are associated with better outcome in acute heart failure: A propensity-score matched study. *Eur J Heart Fail.* (2018) 20:345–54. 10.1002/ejhf.932 28849606

[2] Crespo-Leiro MG Anker SD Maggioni AP Coats AJ Filippatos G Ruschitzka F European society of cardiology heart failure long-term registry (ESC-HF-LT): 1-year follow-up outcomes and differences across regions. *Eur J Heart Fail.* (2016) 18:613–25. 10.1002/ejhf.566 27324686

[3] Acanfora D Scicchitano P Acanfora C Maestri R Goglia F Incalzi RA Early initiation of sacubitril/valsartan in patients with chronic heart failure after acute decompensation: A case series analysis. *Clin Drug Investig.* (2020) 40:493–501. 10.1007/s40261-020-00908-4 32193801

[4] Martin CA Lambiase PD. Pathophysiology, diagnosis and treatment of tachycardiomyopathy. *Heart.* (2017) 103:1543–52. 10.1136/heartjnl-2016-310391 28855272 PMC5629945

[5] Hellawell JL Margulies KB. Myocardial reverse remodeling. *Cardiovasc Ther.* (2012) 30:172–81. 10.1111/j.1755-5922.2010.00247.x 21108773

[6] Michowitz Y Kronborg MB Glikson M Nielsen JC. The ‘10 commandments’ for the 2021 ESC guidelines on cardiac pacing and cardiac resynchronization therapy. *Eur Heart J.* (2021) 42:4295. 10.1093/eurheartj/ehab699 34586378

[7] D’Auria F Polito MV Vitulano G Ciccarelli M De Rosa R Gigantino A Predictors of left ventricular reverse remodeling in patients with chronic heart failure. *J Cardiovasc Med.* (2018) 19:465–9. 10.2459/JCM.0000000000000679 29952847

[8] Chioncel O Lainscak M Seferovic PM Anker SD Crespo-Leiro MG Harjola VP. Epidemiology and one-year outcomes in patients with chronic heart failure and preserved, mid-range and reduced ejection fraction: An analysis of the ESC heart failure long-term registry. *Eur J Heart Fail.* (2017) 19:1574–85. 10.1002/ejhf.813 28386917

[9] Kimura Y Okumura T Morimoto R Kazama S Shibata N Oishi H A clinical score for predicting left ventricular reverse remodelling in patients with dilated cardiomyopathy. *ESC Heart Fail.* (2021) 8:1359–68. 10.1002/ehf2.13216 33471966 PMC8006712

[10] Chen T Guestrin C. XGBoost: A scalable tree boosting system. *The 22nd ACM SIGKDD International Conference.* New York, NY: ACM (2016).

[11] Sagi O Rokach L. Approximating XGBoost with an interpretable decision tree. *Inf Sci.* (2021) 572:522–42.

[12] Wang R Luo W Liu Z Liu W Liu C Liu X Integration of the Extreme Gradient Boosting model with electronic health records to enable the early diagnosis of multiple sclerosis. *Mult Scler Relat Disord.* (2021) 47:102632. 10.1016/j.msard.2020.102632 33276240

[13] Podder P Bharati S Mondal M Kose U. Application of machine learning for the diagnosis of COVID-19. In: Kose U Gupta D editors. *Data Science for COVID-19.* Amsterdam: Elsevier (2021) p. 175–94.

[14] Montomoli J Romeo L Moccia S Bernardin S Migliorelli L Berardini D. Machine learning using the extreme gradient boosting (XGBoost) algorithm predicts 5-day delta of SOFA score at ICU admission in COVID-19 patients. *J Intens Med.* (2021) 1:110–6. 10.1016/j.jointm.2021.09.002 PMC8531027 36785563

[15] Hsiao YW Tao CL Chuang EY Lu TP. A risk prediction model of gene signatures in ovarian cancer through bagging of GA-XGBoost models. *J Adv Res.* (2020) 30:113–22. 10.1016/j.jare.2020.11.006 34026291 PMC8132202

[16] Shtar G Rokach L Shapira B Nissan R Hershkovitz A. Using machine learning to predict rehabilitation outcomes in post-acute hip fracture patients. *Arch Phys Med Rehabil.* (2021) 102:386–94. 10.1016/j.apmr.2020.08.011 32949551

[17] Momenzadeh N Hafezalseheh H Nayebpour MR Fathian M Noorossana R. A hybrid machine learning approach for predicting survival of patients with prostate cancer: A SEER-based population study. *Inform Med Unlocked.* (2021) 27:100763.

[18] Shinbane JS Wood MA Jensen DN Ellenbogen KA Fitzpatrick AP Scheinman MM. Tachycardia-induced cardiomyopathy: A review of animal models and clinical studies. *J Am Coll Cardiol.* (1997) 29:709–15. 10.1016/s0735-1097(96)00592-x 9091514

[19] Fanelli G Dantone M Gall J Fossati A Gool L. Random forests for real time 3D face analysis. *Int J Comput Vis.* (2013) 101:437–58.

[20] Cannata A Manca P Nuzzi V Gregorio C Artico J Gentile P Sex-specific prognostic implications in dilated cardiomyopathy after left ventricular reverse remodeling. *J Clin Med.* (2020) 9:2426. 10.3390/jcm9082426 32751220 PMC7464387

[21] Wilcox JE Fang JC Margulies KB Mann DL. Heart failure with recovered left ventricular ejection fraction: JACC scientific expert panel. *J Am Coll Cardiol.* (2020) 76:719–34. 10.1016/j.jacc.2020.05.075 32762907

[22] Levy RL White PD White PD Stroud WD Hillman CC. Transient tachycardia; prognostic significance alone and in association with transient hypertension. *Med Press Egypt.* (1946) 38:207–12. 20278752

[23] Kannel WB Kannel C Paffenbarger RS Jr. Cupples LA. Heart rate and cardiovascular mortality: The framingham study. *Am Heart J.* (1987) 113:1489–94. 10.1016/0002-8703(87)90666-1 3591616

[24] Tavazzi L Senni M Metra M Gorini M Cacciatore G Chinaglia A Multicenter prospective observational study on acute and chronic heart failure: One-year follow-up results of IN-HF (Italian network on heart failure) outcome registry. *Circ Heart Fail.* (2013) 6:473–81. 10.1161/CIRCHEARTFAILURE.112.000161 23476054

[25] Malhotra R Johnstone C Halpern S Hunter J Banerjee A. Duration of motor block with intrathecal ropivacaine versus bupivacaine for caesarean section: A meta-analysis. *Int J Obstet Anesth.* (2016) 27:9–16. 10.1016/j.ijoa.2016.03.004 27106206

[26] Tadic M Pieske-Kraigher E Cuspidi C Morris DA Burkhardt F Baudisch A Right ventricular strain in heart failure: Clinical perspective. *Arch Cardiovasc Dis.* (2017) 110:562–71. 10.1016/j.acvd.2017.05.002 28669483

[27] Motoki H Borowski AG Shrestha K Hu B Kusunose K Troughton RW Right ventricular global longitudinal strain provides prognostic value incremental to left ventricular ejection fraction in patients with heart failure. *J Am Soc Echocardiogr.* (2014) 27:726–32. 10.1016/j.echo.2014.02.007 24679740

[28] Yuan H Fan XS Jin Y He JX Gui Y Song LY Development of heart failure risk prediction models based on a multi-marker approach using random forest algorithms. *Chin Med J.* (2019) 132:819–26. 10.1097/CM9.0000000000000149 30829708 PMC6595865

[29] Pérez-Rodon J Galve E Pérez-Bocanegra C Soriano-Sánchez T Recio-Iglesias J. A risk score to predict the absence of left ventricular reverse remodeling: Implications for the timing of ICD implantation in primary prevention. *J Cardiol.* (2018) 71:505–12. 10.1016/j.jjcc.2017.10.019 29183646

[30] Kubanek M Sramko M Maluskova J Kautznerova D Weichet J Lupinek P Novel predictors of left ventricular reverse remodeling in individuals with recent-onset dilated cardiomyopathy. *J Am Coll Cardiol.* (2013) 61:54–63. 10.1016/j.jacc.2012.07.072 23287372

[31] Fornwalt BK Sprague WW BeDell P Suever JD Gerritse B Merlino JD Agreement is poor among current criteria used to define response to cardiac resynchronization therapy. *Circulation.* (2010) 121:1985–91. 10.1161/CIRCULATIONAHA.109.910778 20421518 PMC2882855

